# Efficient Solar‐Thermal Distillation Desalination Device by Light Absorptive Carbon Composite Porous Foam

**DOI:** 10.1002/gch2.201900003

**Published:** 2019-04-08

**Authors:** Gyoung Gug Jang, James William Klett, Joanna McFarlane, Anton Ievlev, Kai Xiao, Jong K. Keum, Mina Yoon, Piljae Im, Michael Z. Hu, James E. Parks

**Affiliations:** ^1^ Energy and Transportation Science Division Oak Ridge National Laboratory Oak Ridge TN 37831 USA; ^2^ Materials Science and Technology Division Oak Ridge National Laboratory Oak Ridge TN 37831 USA; ^3^ Isotope and Fuel Cycle Technology Division Oak Ridge National Laboratory Oak Ridge TN 37831 USA; ^4^ Center for Nanophase Materials Science Oak Ridge National Laboratory Oak Ridge TN 37831 USA

**Keywords:** distillation, graphite foam, solar desalination, superhydrophobicity

## Abstract

Solar‐thermal driven desalination based on porous carbon materials has promise for fresh water production. Exploration of high‐efficiency solar desalination devices has not solved issues for practical application, namely complicated fabrication, cost‐effectiveness, and scalability. Here, direct solar‐thermal carbon distillation (DS‐CD) tubular devices are introduced that have a facile fabrication process, are scalable, and use an inexpensive but efficient microporous graphite foam coated with carbon nanoparticle and superhydrophobic materials. The “black” composite foam serving as a solar light absorber heats up salt water effectively to produce fresh water vapor, and the superhydrophobic surface of the foam traps the liquid feed in the device. Two proof‐of‐principle distillation systems are adopted, i.e., solar still and membrane distillation and the fabricated devices are evaluated for direct solar desalination efficiency. For the solar still, nanoparticle and fluorosilane coatings on the porous surface increase the solar energy absorbance, resulting in a solar‐steam generation efficiency of 64% from simulated seawater at 1 sun. The membrane distillation demonstrates excellent vapor production (≈6.6 kg m^‐2^ h^‐1^) with >99.5% salt rejection under simulated 3 sun solar‐thermal irradiation. Unlike traditional solar desalination, the adaptable DS‐CD can easily be scaled up to larger systems such as high‐temperature tubular modules, presenting a promising solution for solar‐energy‐driven desalination.

Global scarcity of fresh water has driven intensive research in solar desalination to provide clean water directly using processes with minimum environmental impact. Despite the enormous potential of solar energy, the drawbacks in terms of cost‐effectiveness and desalination efficiency have been huge barriers to utilization. In the traditional approach as in solar still and solar chimney, a solar collector is coupled with a distillation mechanism and the process is carried out in one simple cycle.^1^ However, in the traditional approach employing direct solar desalination, large‐scale deployment is limited by expensive cost and losses in system efficiency, for example, estimated production rate 3–12 kg m^−2^ per day,^1^ where the low efficiency originates from the heat and mass transfer occurring during evaporation and condensation. Hence, indirect solar desalination using photovoltaic (PV) panels and other traditional desalination technologies, for example, distillation and reverse osmosis (RO) have been developed.^2^ The conversion efficiency of PV is, however, still low, less than 15% for electricity generation. Recent development of various nanostructured materials, including plasmonic nanoparticles, porous graphene, and ultra‐black absorbers composited as an interfacial evaporator, have made it possible to transfer solar‐heat directly to generate water vapor with high efficiency.^3^, ^4^, ^5^, ^6^, ^7^, ^8^, ^9^, ^10^ In order to achieve efficient solar interfacial evaporation, two key elements are required: 1) efficient solar absorption using blackbody materials and 2) efficient heat management using thermal localization structures as in nanocapillary channels with thermally insulating layers or containers. It has been shown that the engineered interfacial solar evaporators using the two aforementioned aspects can double the light‐to‐heat conversion efficiency, increasing pure water production from 0.5 to 1.6 kg m^−2^ h^−1^ (i.e., 27–86% of the conversion efficiency) under 1 sun illumination (=1 kW m^−2^).^3^, ^4^, ^5^, ^6^, ^7^, ^8^, ^9^, ^10^ Without a thermal insulating layer, on the other hand, the efficiency of blackbody material was found to be low (e.g., 30–45%) because of heat transfer and therefore heat loss to bulk water.^11^, ^12^ It has recently been shown that the conversion efficiency could be improved to >90% using a hierarchically nanostructured system at 1 sun irradiation.^13^ However, the current interfacial solar steam generators require flat implementation geometry and, thus, produce much less fresh water than energy‐intensive traditional distillation and membrane distillation systems (e.g., 1–30 kg m^−2^ h^−1^) with tubular geometry.^14^, ^15^, ^16^ Although it has been demonstrated that the flux can be increased by using concentrated solar power (10 kW m^−2^ or higher), features of these interfacial nanostructures, such as being able to float on water, are best suited to provide efficient solar vapor generation under 1 sun or natural daylight.^13^ As these nanostructures are to be extended to large scales, further understanding of solar thermal conversion and heat and mass transfer is needed to address practical problems such as scalability, module design, compatibility with the existing facilities, and fabrication cost.

Membrane distillation (MD) is a thermally driven separation using a hydrophobic microporous polymer membrane. The production rate can be increased by maximizing the membrane surface area in a small configuration as in a spiral wound module. The hydrophobicity of the membrane helps trapping liquid feed on the upstream side, while the microporous structure allows water vapor permeation through the membrane.^15^, ^16^ MD is considered to be a cost‐effective desalination technique because of lower operation temperatures and narrower vapor space than multi‐effect distillation, lower operation pressure than RO, high salt rejection rate (99.9%), with no limitations imposed by high osmotic pressure or concentration gradient.^15^ Still, thermal energy requirements are high in currently used MD desalination processes, but the use of waste heat and renewable energy could enable large cost savings.^15^ Considering these advantages of MD, a direct solar‐driven MD system using nanophotonics was developed, with a thermal management system that can be scaled up to a system with higher operation temperatures and water production rates.^17^ However, this approach exhibited relatively low conversion efficiency (i.e., 21%) under 1 sun irradiation due to the permeation vapor flux of the polymer membrane, being dominated by small pores, porosity, and thickness.

Having a planar membrane module system for capturing sun light limits flux and efficiency. On the contrary, concentrated solar power (CSP) uses mirrors to reflect and concentrate sunlight onto a small receiver. For example, in a parabolic trough concentrator, a linear parabolic reflector concentrates light onto a receiver positioned along the reflector's focal line.^18^ The receiver is a tube positioned directly above the middle of the parabolic mirror and filled with a working fluid. The reflector follows the sun during daylight hours by tracking along a single axis. The working fluid is heated up to 150–350 °C as it flows through the receiver and becomes a heat source for power generation. We consider the parabolic trough system a candidate CSP technology for adopting direct solar–thermal distillation. In this case, a tubular module is required. We believe tubular modulization can effectively increase heat transfer and vapor generation efficiency.

We report the first direct solar–thermal carbon distillation (DS‐CD) tubular devices based on a porous carbon nanoparticle (NP) composite graphite foam (GF) coated with a superhydrophobic (SP) material. Inspired by the polymeric MD configuration, solar still, and scalable thermal‐conductive GF, we demonstrated two DS‐CD desalination devices: 1) a solar distillation membrane and 2) a solar distillation heat exchanger. In the solar distillation membrane, a black porous GF outer shell absorbs solar energy and the absorbed heat transfers to the salt water inside the tube producing fresh water vapor. The microporous structure in the skin layer of the membrane allows water vapor to permeate through the membrane and trap the bulk salt liquid within the SP surface coated membrane. In the solar distillation heat exchanger, the black GF beneath a salt water layer absorbs solar energy to heat the salt water to produce fresh water vapor and the SP surface of the porous NP layer traps the liquid feed on the surface (**Figure** **1**).

**Figure 1:**
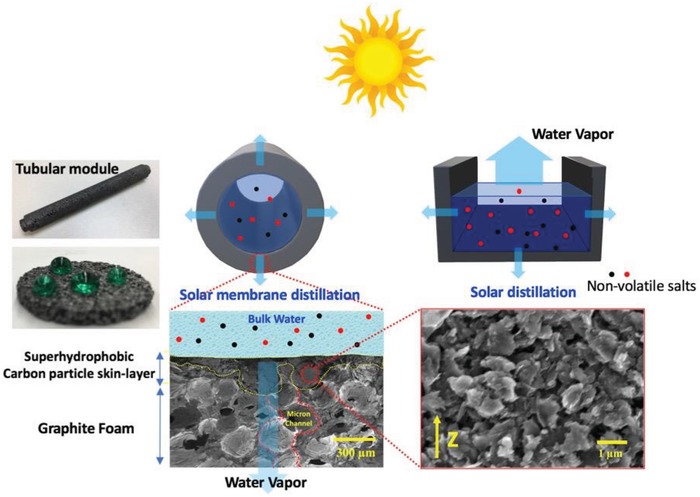
Graphical illustration of two direct solar–thermal carbon distillation (DS‐CD) systems made from SP‐coated blackbody carbon NP composite porous GF. The cross section of the nanoparticle skin layer at the inset of SEM (red line) shows the porosity of the vapor channel.

GF is a black 3D structural material in which the crystallized graphite ligaments formed in interconnected open cells with pores.^19^, ^20^, ^21^ The GF has a low average bulk density of 0.2–0.6 g cm^−3^, compression strength and modulus of ≈5 MPa and ≈410 MPa, respectively. Moreover, it has a bulk thermal conductivity as high as >150 W m^−1^ K^−1^ with controllable pore and high porosity (≈80%).^19^, ^20^, ^21^ The strong, lightweight GF enables the development of various structured modules such as plates, tubes and chambers using simple cutting, drilling, and machining. Scanning electron microscopy (SEM) showed that cavities and pores of the cell formed micron‐scale channels that had widths ranging from tens to hundreds of microns (highlighted by the red broken line in Figure 1). Also, the wall structure exhibited cracked internal surfaces for additional vapor channels. Because of its micro/macropores and nanotextured surfaces, GF can be used as a template (here, a membrane support for solar energy absorption and heat transportation), and functional materials can be introduced inside the cells and interior surfaces to enhance their performance and thus enable highly functionalized devices.

To evaluate pore size and solar‐absorbance control, a graphite NP membrane was deposited onto the GF by slurry coating with graphite NPs (0.5–1 µm) and phenolic resin, followed by thermal curing for 1 h at 300 °C. The particle coating was applied to the surface of the foam with pores and was squeezed into the open cells on the surface. The top and cross section of the coating layer in **Figures** 1 and **2**d exhibit the open pores with the diameter ranging from ≈300 to ≈800 nm. To acquire hydrophobic water‐trapping capability, the GF substrates were then immersed in a solution of fluorosilane that is deposited on the substrate to reduce the surface free energy. The fluorosilane groups were hydrolyzed and hydrogen‐bonded to the GF surface through interaction with the molecular layers of adsorbed water (Figures S2 and S3, Supporting Information). As shown in Figure 2a, after hydrophobic modification, the GF substrate exhibited superhydrophobicity with the water contact angle of >150°. The GF blocks were cut with an electric saw to create a hydrophobic micron‐scale roughened surface at the cell walls and pores, resulting in air pockets associated with superhydrophobicity. Water droplets easily rolled off the surface without adhesion, exhibiting Cassie's wetting behavior.^22^ Under the Cassie's wetting state, contact between the water and solid substrate surface is minimized, which is considered to be unfavorable for heat transfer from the GF to the water. Floating wettable GF substrates produced more water vapor than the SP‐coated (non‐wettable) GF substrate (Figure S4, Supporting Information). However, the non‐wetting behavior is a critical factor in the design of a distillation heat exchanger and an MD membrane for fresh vapor production, along with bulk salt water leakage prevention and long‐term anti‐fouling performance.

**Figure 2:**
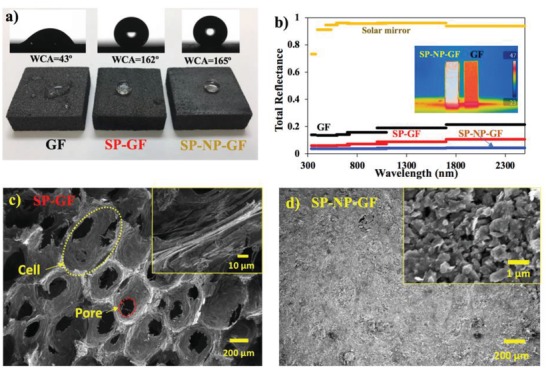
Characterization of the SP‐coated porous‐conductive GF. a) GF, SP‐GF, and SP carbon NP composite foam (SHP‐NP‐GF). b) Total reflectance spectra of GFs. Inset is IR thermal image of their heat absorption properties under solar–thermal irradiation. c) Scanning electron microscope (SEM) image of SP‐GF. d) SEM image of SP‐NP‐GF.

In the pore‐size controlled GF (SP‐NP‐GF: ≈500 nm of open pores), the membrane also exhibited superhydrophobicity (>150°) originating from the rough surface of the NP layer. Figure 2b shows that the intrinsic GF has a total reflectance of 0.155 (light absorption [*A*] = 84.5%) over a wavelength range of 330–2500 nm (typical solar radiation). Note that, when the fluorosilane molecules were coated on the foam surface, the surface became darker, reducing the total reflectance to 0.068 (*A* = 93.2%). The self‐assembled thin molecular coating changed the reflective index of the surface, increasing the solar absorption. The fluorosilane coating on the surface of carbon NP membrane further reduced the total reflectance to 0.036 (*A* = 96.4%) since the molecular coating created a surface with nano‐scale roughness and hence the light absorption was enhanced. The intrinsic NP membrane showed 0.052 total reflectance (*A* = 94.8%). Under simulated solar–thermal irradiation, SP‐NP‐GF absorbed more photons than untreated GF (inset in Figure 2b).

Three solar distillation systems were evaluated for solar–thermal evaporation performance: 1) open‐vessel distillation (OD), 2) flow channel distillation (FD), and 3) membrane distillation (MD) (**Figure** **3**). A GF vessel with a lid (5 mm thick, 12 mm deep, inner diameter 60 mm, and outer diameter 70 mm) was machined and the internal cavity was SP‐coated. For MD desalination, the SP‐GF membrane vessel was filled with simulated sea water (3.5 wt% NaCl, 20 g) and the lid was sealed to make a watertight vessel. The water layer was ≈0.7 cm. The membrane vessel was inverted and the bottom of the vessel was exposed to various simulated solar–thermal irradiation. An infrared (IR) camera and a thermocouple were used to probe the temperature of the vessel (inside and top surface) and a control sample. The temperature inside increased similarly to the top surfaces because of the high thermal conductivity of the vessel (Figure S5, Supporting Information). After the MD measurement for an SP‐GF vessel with ≈200 µm pores (Figure 2c), the inside of the GF chamber and the lid were coated with carbon NPs (BET = 250 ± 25 m^2^ g^−1^) and flurosilane to investigate the effects of pore size in an MD system with ≈0.5 µm pores (Figure 2d). Then the lid was drilled to fabricate vapor flow channels (1.6 mm diameter, *N* = 200) for FD desalination.

**Figure 3:**
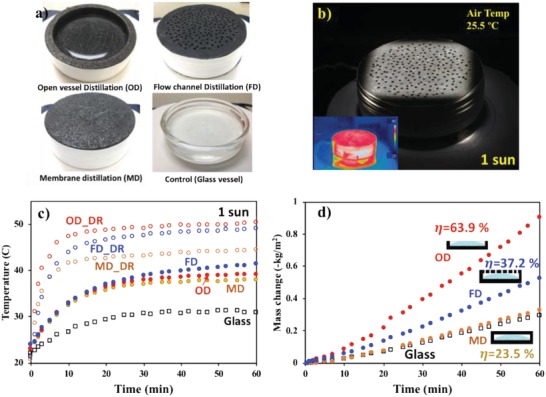
a) Various distillation devices for solar desalination. b) Solar evaporation of flow channel distillation (FD) under 1 sun irradiation. The air temperature under irradiation was measured at 5 cm above the chamber. c) The temperature rise of the distillation devices over time. The DR (dry‐run) in (c) denotes experiment for the empty chambers under 1 sun. d) Evaporation mass loss of salt water (3.5 wt% of NaCl) under 1 sun irradiation. All experiments were conducted at an ambient temperature of 21 °C.

Two types of simulated solar–thermal desalination experiments were carried out to evaluate the DS‐CD desalination performance: 1) irradiation by a nominal solar simulator (solar DS‐CD) with an intensity of 1 kW m^−2^ (one sun), and 2) irradiation by a heat lamp (solar–thermal DS‐CD) with various visible light and IR intensities. To represent ambient temperature condition in hot, arid areas, we designed a solar–thermal simulator to mimic solar heat radiation using an incandescent heat lamp (2700 K lamp). **Figure** **4**a presents the experimental setup for solar–thermal DS‐CD measurement. The concentrated solar intensity (e.g., 0.87–3 sun intensity) was calibrated while maintaining the distance between the midpoint of the outer surface of the lamp and the vessel surface. With solar–thermal simulation, the ambient temperature 5 cm above the GF chamber was ≈37.2 °C at 0.87 sun. The solar intensity calibration details are available in the supporting information (Figure S6).

**Figure 4:**
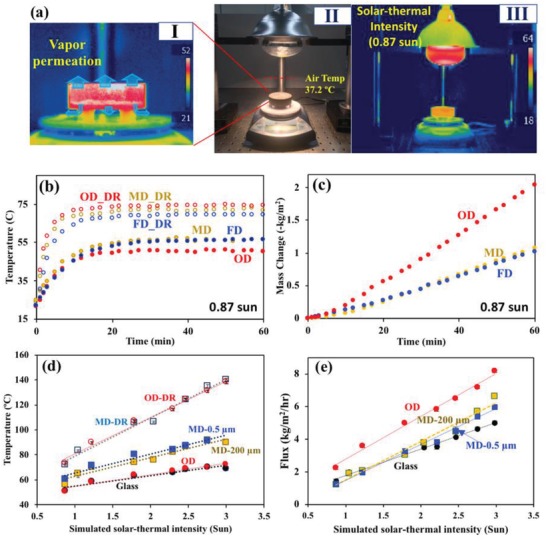
a) IR and optical images of evaporation in a DS‐CD system under simulated solar–thermal irradiation (0.87 sun) by a heat lamp. The air temperature under irradiation was measured at 5 cm above the chamber. b) Temperature rise of the distillation devices over time under 0.87 sun solar–thermal irradiation. The dry‐run (DR) is the temperature rise of the empty vessels under the solar–thermal irradiation. c) The corresponding evaporation mass loss of salt water (3.5 wt% NaCl). d) The average temperature profiles for 30–60 min of OD and MD devices compared with open bulk water in a glass vessel as a control test under different solar–thermal irradiations. e) The permeate flux of OD and MD devices under different solar–thermal illuminations. The dotted lines are linear trend fittings. Error bars are the standard deviations in the mean value (*N* = 3). The error bars may not be seen because of the low values (<2%).

Having confirmed the photothermal effect, we measured the evaporation rate of simulated sea water (3.5 wt% salinity). The simulated solar light with an intensity of 1 kW⋅m^−2^ shone directly onto the top surface of each distillation vessel. For the dry run (without salt‐water), the OD vessel with an SP‐NP‐GF layer was heated to ≈47 °C in 10 min; for OD desalination, it was heated to ≈37.9 °C in 30 min, resulting in an effective permeate flux of 1.02 kg m^−2^ h^−1^ (calculated from 0.5 to 1 h) with a vapor‐generation efficiency of 63.9% (Figure 3d). The vapor‐generation efficiency was calculated based on the following relationship^6^, ^13^

$$\eta =\dot{m}h_{\mathrm{LV}}/C_{\mathrm{opt}}P_{0}$$

where $\dot{m}$ refers to the mass flux, *h*_LV_ to the total liquid‐vapor phase change enthalpy including the sensible heat, *P*_0_ is the nominal solar irradiation value of 1 kW m^−2^, and *C*_opt_ represents the optical concentration. We assumed that the OD thermal driving force (i.e., *T*_dry‐run_ *‐T*_OD_ for 30–60 min) for water evaporation was Δ11.2 °C. For MD desalination, the porous membrane vessel (200 µm) allowed water vapor permeation from both top and bottom surfaces, even though only the top surface was irradiated. In this MD system, the effective permeate flux was determined by the mass change per inside top surface area. The water loss by evaporation under 1 sun light irradiation was 0.34 kg m^−2^ h^−1^ with an efficiency of 23.5%. The vapor generation efficiency could be enhanced by adopting different MD configurations (e.g., direct contact MD), an additional driving force (e.g., vacuum) and a condensation system.^23^ Our system is similar to an air‐gap MD (1–5 kg m^−2^ h^−1^) which has >10 times lower permeation flux than direct‐contact MD (5–30 kg m^−2^ h^−1^).^16^ The MD thermal driving force for water evaporation was Δ6.2 °C. For the FD system, the effective permeate flux was 0.59 kg m^−2^ h^−1^ with a solar–thermal conversion efficiency of 37.2% and a thermal driving force of Δ8.1 °C. As a control, 20 g of simulated seawater was heated in a glass beaker with the same diameter of the GF vessel. The control water was heated to 30 °C with an efficiency of 21.7% under the same conditions. Note that the vapor permeate flux and heat management of the DS‐CD was effectively controlled by the modulization (see another module system and heat management in Figure S7, Supporting Information).

Under concentrated solar–thermal irradiation at 0.87 sun, the OD, MD, and FD, vapor permeation flux increased significantly to 2.26, 1.25, and 1.15 kg m^−2^ h^−1^, respectively, because more IR light was applied to the device surfaces (Figure 4). The high permeation flux of OD was attributed to its higher thermal driving force, Δ23.8 °C, compared with Δ15.8 °C for MD and Δ13.4 °C for FD. It appears that having a separate lid and chamber in the FD module system may have induced heat loss between the gaps at the high‐IR condition. Figure 4d,e shows the temperature and permeate flux profiles as a function of simulated solar–thermal intensity. The temperature of the device chamber and the permeate flux increased as the solar–thermal intensity increased as a result of the increased light and heat absorption. The vapor OD permeate flux was significantly higher than that of the control (glass chamber) and the water production of the other MD system at the intensity range of 0.8–3 sun. The heat driving force of OD was Δ22.9–Δ56.2 °C over the range, while that of MD was Δ11.5–Δ50.3 °C. At an intensity of 3 sun, the MD‐200 µm system showed higher vapor flux than the control glass chamber. The MD performance is associated with better absorption efficiency of the carbon materials at high intensity, resulting from a larger thermal driving force, in agreement with the literature.^6^, ^9^, ^10^, ^24^ A high solar–thermal conversion efficiency of >70% was found at a solar intensity of >3 sun. The MD permeate performance of 6.6 kg m^−2^ h^−1^ at 3 sun intensity is comparable to that of the polymer air gap MD membrane (e.g., 1–5 kg m^−2^ h^−1^ for seawater desalination).^16^ At the same condition, OD exhibited the highest vapor production with 8.8 kg m^−2^ h^−1^.

Control of the pore size in the membrane is a critical factor for stable MD desalination. In traditional MD, membrane hydrophobicity, pore size, porosity, and thickness all play significant roles in the permeation flux, selectivity, and energy efficiency.^14^ As discussed, we controlled the pore size (≈200 µm) of the SP‐GF membrane by filling the open cells (≈500 µm) with carbon NPs. The GF membrane with pore sizes controlled at 0.6 ± 0.5 µm (MD‐0.5 µm) exhibited a lower permeate flux. However, the vapor flux reduction (7–10%) through MD‐0.5 µm appeared to be smaller than that through analogous polymeric air‐gap MD membranes (i.e., 40% reduction through a polyvinylidene fluoride membrane with 70% of pore sizes reduction).^25^ A possible explanation for this improvement is on the control and stabilization of the thermal driving force—one of the most important factors in determining the permeate flux in an MD system. For example, conventional MD systems have low vapor flux across the membrane because of “temperature polarization,” and inevitable thermal energy loss related to the removal of latent heat with water evaporation at operation. As a result, the net thermal driving force to the evaporation decreases and, ultimately, the overall efficiency of the process deteriorates. But direct solar–thermal MD has increased throughput across the membrane because the water is continuously heated by constant irradiation.^17^, ^23^ Thus, a decrease in input flow velocity and higher ambient temperature benefit direct solar–thermal MD over traditional MD. Note that the thermal driving force of our MD system did not decrease during the operation (Figure 4b). The temperature of MD‐0.5 µm desalination was 2–5 °C higher than the temperature of MD‐200 µm desalination over the same intensity range. The smaller pore size decreases the mass transfer, while increasing the heat accumulation in the system. The accumulated heat increased the vapor pressure, resulting in greater permeate flux. To simulate a planar membrane module system, the sides and bottom of the MD membrane vessel were sealed with Teflon tape and aluminum foil, leaving the top as the only open surface (Figure S8, Supporting Information). The amount of vapor permeating the MD system dropped by only 10–20% from the amount passing through the open top‐bottom membrane system, even with a factor of two reduction in membrane surface area. The temperature inside the chamber was significantly higher because of the reduced porosity and membrane surface area. The increased temperature of the open top system (≈10 °C higher than the open top‐bottom membrane system) also increased the vapor pressure of the water inside the vessel, so more water vapor permeation was observed.

The salinity of the vapor permeating from the salt water in the OD, FD, and MD systems was measured by electrical conductivity. A transparent glass chamber was capped on the SP‐NP‐GF devices, and the permeated vapor was observed to condense inside the beaker during irradiation (Figure S9, Supporting Information). The concentrations of Na^+^ ions collected from the condensation were determined to be 0.01–0.017 wt% with 99.5% salt rejection. The salinities of the condensates were below the drinking water standards determined by the World Health Organization (0.1 wt%) and the US environmental Protection Agency (0.05 wt%).^26^ With sufficient time (≈80 min for 20 g of salt water), the system completely dried out and the salt formed a single dried cake (≈0.7 g) at the edge of the cavity. The explanation is that beads of NaCl solution became smaller, the surface tension increased relative to interfacial tension with the SP coating. Thus, the water droplets remained intact rather than leaving a distributed salt layer as occurs with evaporation from an open vessel. This beading may mitigate scaling and potential DS‐CD fouling. To evaluate the durability of the DS‐CD device, the MD‐200 µm system was run for more than 30 cycles, with each cycle sustained for over 1 h. Then the MD‐200 µm vessel was regenerated by annealing at 400 °C in air for 6 h. After annealing, the fluorosilanes on the GF decomposed and the dewetting performance was lost. A new SP‐NP‐GF device was fabricated on the same substrate. The OD and FD systems using the SP‐NP‐GF device exhibited stable evaporation rates for a further 60 cycles. For scalability, a larger tubular SP‐GF membrane (length 30 cm, 3.5 cm, and 1 cm) was fabricated and evaluated (Figure S10, Supporting Information). For an existing solar–thermal distillation system that uses electricity from photovoltaics to generate heat, the cost of thermal energy is $ 0.4–1.4 per cubic meter, 45–74% of the levelized cost of water.^27^ The renewable, scalable, durable DS‐CD device is expected to significantly reduce thermal energy requirements and thus reduce costs.

Using GF functionalized with low‐surface‐energy flurocarbonate and carbon NPs, we fabricated SP carbon NP composite GF distillation structures that demonstrated effective solar absorption of over 96% and 64% solar–thermal conversion efficiency respectively. The efficient solar absorption, controllable heat management, and porous nature of this structure enables estimated direct‐solar desalination performance of ≈8.8 kg m^−2^ h^−1^ of permeate flux and ≈99.5% salt rejection under simulated concentrated solar–thermal irradiation. We anticipate that further surface modification (e.g., surface roughness and enhanced light absorptive coating) and module optimization (e.g., skin layer thickness, pore size, and integrated platform) of DS‐CD will improve the vapor production performance. Unlike most existing nanostructured desalination strategies for interfacial solar evaporators, our SP‐NP‐GF based DS‐CS device can be scaled into a tubular module for a distillation system. A scaled system may provide an approach for mass production of fresh water adapted to work with CSP technologies. Porous‐conductive composite GF with durable materials and scalable fabrication could therefore provide a practical solution for solar desalination with a minimal carbon footprint that can also be employed in many other separation applications, such as wastewater treatment.

## Experimental Section

*Materials*: GFs with various pore sizes were acquired from Koppers Inc. and CFOAM, LLC. The foam was made from the heat‐treated PI Mesophase Pitch, sieved with 250 mesh, with 7% concentrations of the Ground Foam Graphene platelets added to prepare tubes and chambers.^28^ Graphite particles (grade 4827, 225–275 m^2^ g^−1^) were purchased from Asbury Graphite Mills Inc. and Penolic resin (Durite SC1008) was purchased from Hexion Specialty Chemicals. (Heptadecafluoro‐1,1,2,2‐tetrahydrodecyl) trichlorosilane was purchased from Gelest. Sodium chloride was purchased from Mallinckrodts Chemicals.

*Preparation of photothermal SP carbon membrane*: The GFs were machined with an electrical saw and drills for tubes and chambers. Fluorosilane molecules were then deposited on the GF tube and chamber via solution immersion (0.1 wt% in Hexane) overnight. After the fluorosilane coating, the GF chamber was oven‐dried at 100 °C for 1 h. The GF chamber was rinsed with copious amounts of water and ethanol to remove unbounded silanes. For the pore‐controlled GF membrane, graphite particles and phenolic resins were mixed at weight ratio of 2:1. The particle slurry was coated on the membrane chamber and its lid with a wood stick knife. After drying overnight, the coated GF chamber was thermally cured at 300 °C for 1 h.

*Material Characterization*: Scanning electron microscopy was carried out using a field emission scanning electron microanalyzer (Merlin, Carl Zeiss AG). Water contact angles were measured by an optical tensiometer (OneAttension, Biolin Scientific). Specular and total reflectance from the 330 to the 2500 nm spectra region was measured by a portable reflectometer (410‐Solar, Surface Optics Corporation). The absorption (*A*) efficiency was then calculated by *A* = 1−R−T, where R and T are reflectance and transmission efficiency, respectively.^29^ The transmission efficiency was not considered in the thick GF system (thickness = 5 mm). The surface chemistry of coated samples was measured by time‐of‐flight secondary ion mass spectrometry. The solar irradiation processing was under an illumination of 100 mW cm^−2^ AM 1.5 G solar spectrum using a solar simulator (Radiant Source Technology, 300 W, Class A). The light intensity was calibrated using a NIST‐certified silicon reference cell.

## Conflict of Interest

The authors declare no conflict of interest.

## Supporting information

Supplementary
