# Supplementary material for: Efficient Solar‐Thermal Distillation Desalination Device by Light Absorptive Carbon Composite Porous Foam
Source: Glob Chall. 2019 Apr 8;3(8):1900003. doi: 10.1002/gch2.201900003 (PMC6686170; doi:10.1002/gch2.201900003)
Supplement: Supplementary file 1 — Supplementary [file GCH2-3-1900003-s001.pdf]

Copyright WILEY-VCH Verlag GmbH & Co. KGaA, 69469 Weinheim, Germany, 2019.

Global Challenges
Open Access

Supporting Information

for *Global Challenges,* DOI: 10.1002/gch2.201900003

Efficient Solar-Thermal Distillation Desalination Device by Light Absorptive Carbon Composite Porous Foam

Gyoung Gug Jang, James William Klett, Joanna McFarlane, Anton Ievlev, Kai Xiao, Jong K. Keum, Mina Yoon, Piljae Im, Michael Z. Hu, and James E. ParksII*

Supporting Information

Efficient solar-thermal distillation desalination device by light absorptive composite porous graphite foam

Gyoung Gug Jang[1*], *James William Klett*[2], *Joanna McFarlane*[3], *Anton Ievlev*[4], *Kai Xiao*[4], *Jong K. Keum*[4], *Mina Yoon*[4] *Piljae Im*[1], *Michael Z. Hu*[1], *James E. Parks II* [1]

[1] Energy and Transportation Science Division, Oak Ridge National Laboratory (ORNL), Oak Ridge, TN 37831
[2] Materials Science and Technology Division, ORNL
[3] Nuclear Security and Isotope Technology Division, ORNL
[4] Center for Nanophase Materials Science, ORNL

[*]Corresponding author: jangg@ornl.gov

Notice: This manuscript has been authored by UT-Battelle, LLC, under contract DE-AC05-00OR22725 with the US Department of Energy (DOE). The US government retains and the publisher, by accepting the article for publication, acknowledges that the US government retains a nonexclusive, paid-up, irrevocable, worldwide license to publish or reproduce the published form of this manuscript, or allow others to do so, for US government purposes. DOE will provide public access to these results of federally sponsored research in accordance with the DOE Public Access Plan (http://energy.gov/downloads/doe-public-access-plan).

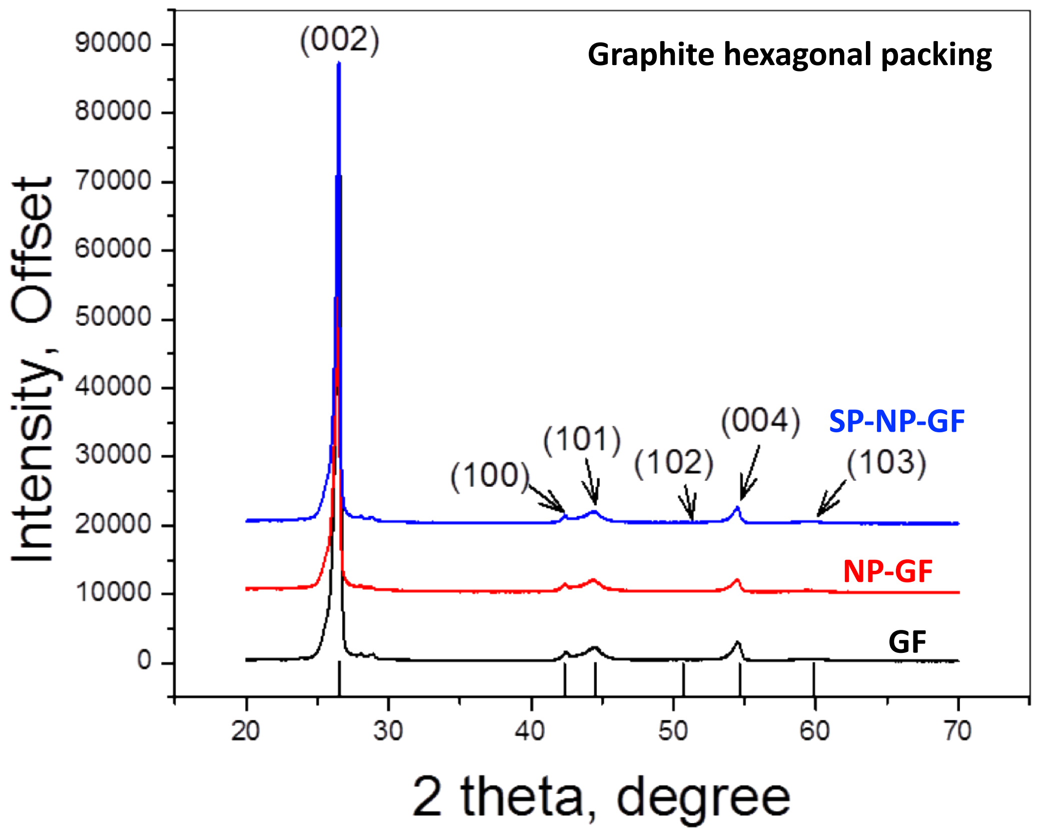

Figure S1. XRD measurements of GF (graphite foam), carbon nanoparticle composite GF (NP-GF), and superhydrophobic material-coated NP-GF (SP-NP-GF) platforms.

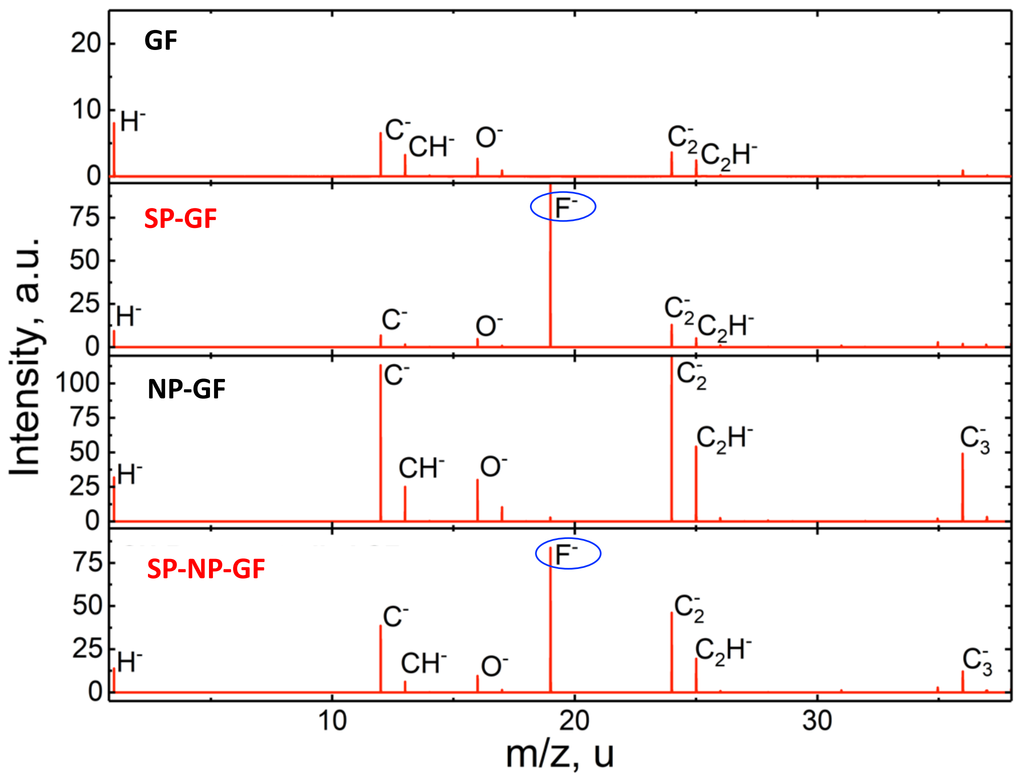

Figure S2. Time of flight–secondary ion mass spectrometry measurements of GF (graphite foam), carbon nanoparticle composite GF (NP-GF), and superhydrophobic material–coated NP-GF (SP-NP-GF) platforms.

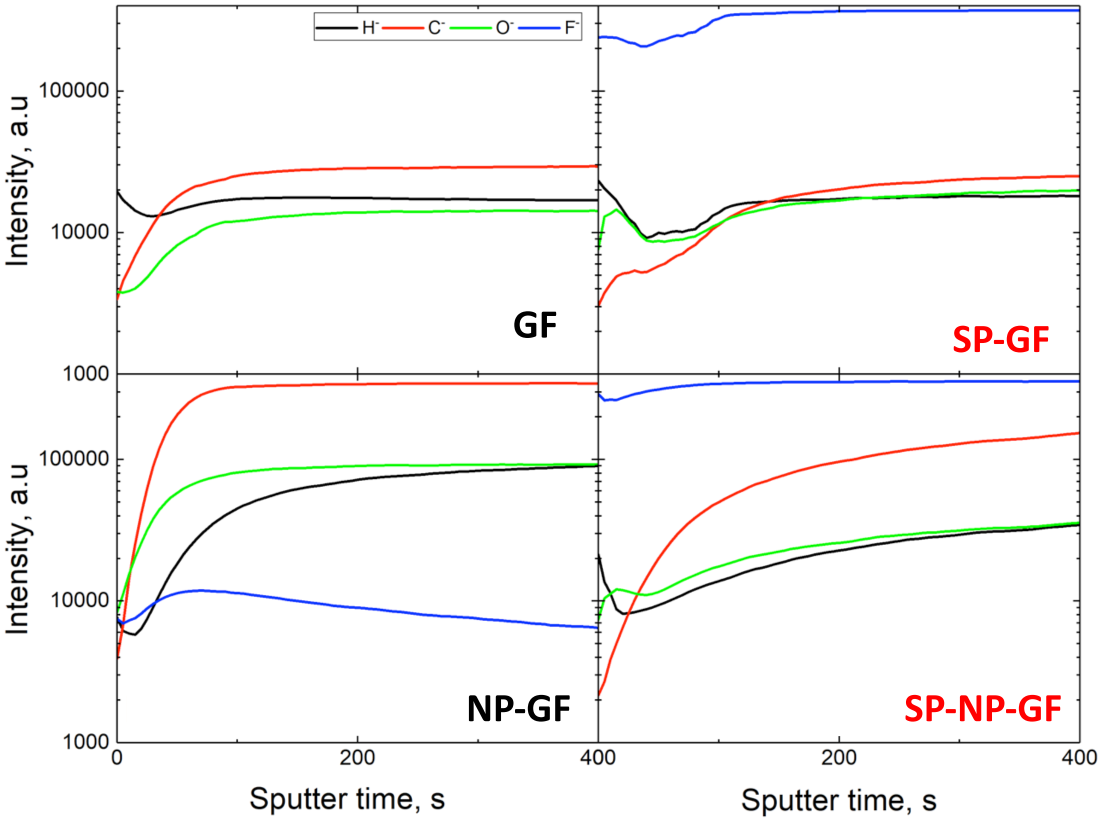

Figure S3. Depth profile of GF (graphite foam), carbon nanoparticle composite GF (NP-GF), and superhydrophobic material–coated NP-GF (SP-NP-GF) platforms.

1. Solar thermal evaporation by Self-floating graphite foam

Figure S4A shows the temperature profile of GF under simulated concentrated solar-thermal irradiation (>3 sun) in an open environment. To characterize this effect and the subsequent convection of the heat to water, a simple test was conducted in which the foam was floated on the surface of salt water in a beaker, placed next to a beaker with no foam. Both beakers were placed on a scale to measure weight loss as a function of time. Simulated solar energy was added to the beakers and the temperature and weight loss reported. Next, the foam was coated with a superhydrophobic (SH) coating on the side facing the water and the experiment was repeated. In both cases, the graphite foam absorbed the simulated solar-thermal energy quickly and reached a temperature of 75°C in about 30 min. The water under the beaker with GF heated faster than the water under the beaker with no foam (see infrared images in Figure S4). In fact, the water in the beaker with no foam reached a substantially lower maximum temperature. The beakers' masses were recorded and revealed that untreated GF exhibited 1.8 times faster vapor production than bulk salt water under the same irradiation condition. The fact that the raw GF had a slightly hydrophilic nature allowed some water to move into the pores, thus increasing the water contact surface area and resulting in an enhanced evaporation rate—in addition to its enhancement of the heating of the water. The beaker with SP-coated GF also exhibited improved vapor production compared with the beaker with no GF, but it was not substantial because the SH tendency of the GF prevented wetting of the foam and a lower GF surface/water contact area.

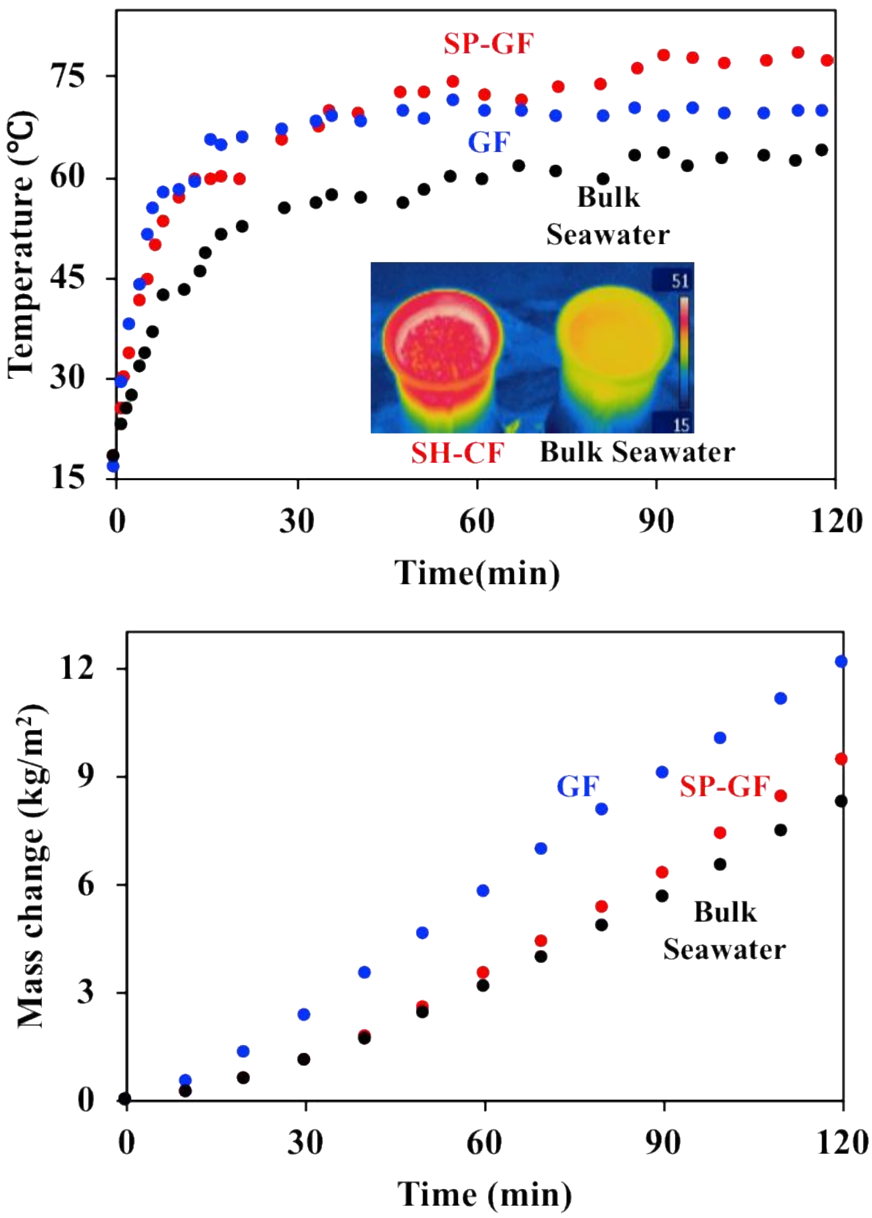

Figure S4. (Top) The temperature profile of self-floating GF substrates on the surface of salt water (3.5 wt %, 100 g) under concentrated solar-thermal (~estimated 3-sun) irradiation. (Bottom) The mass loss of salt water assisted by untreated GF and SH-GF substrates.

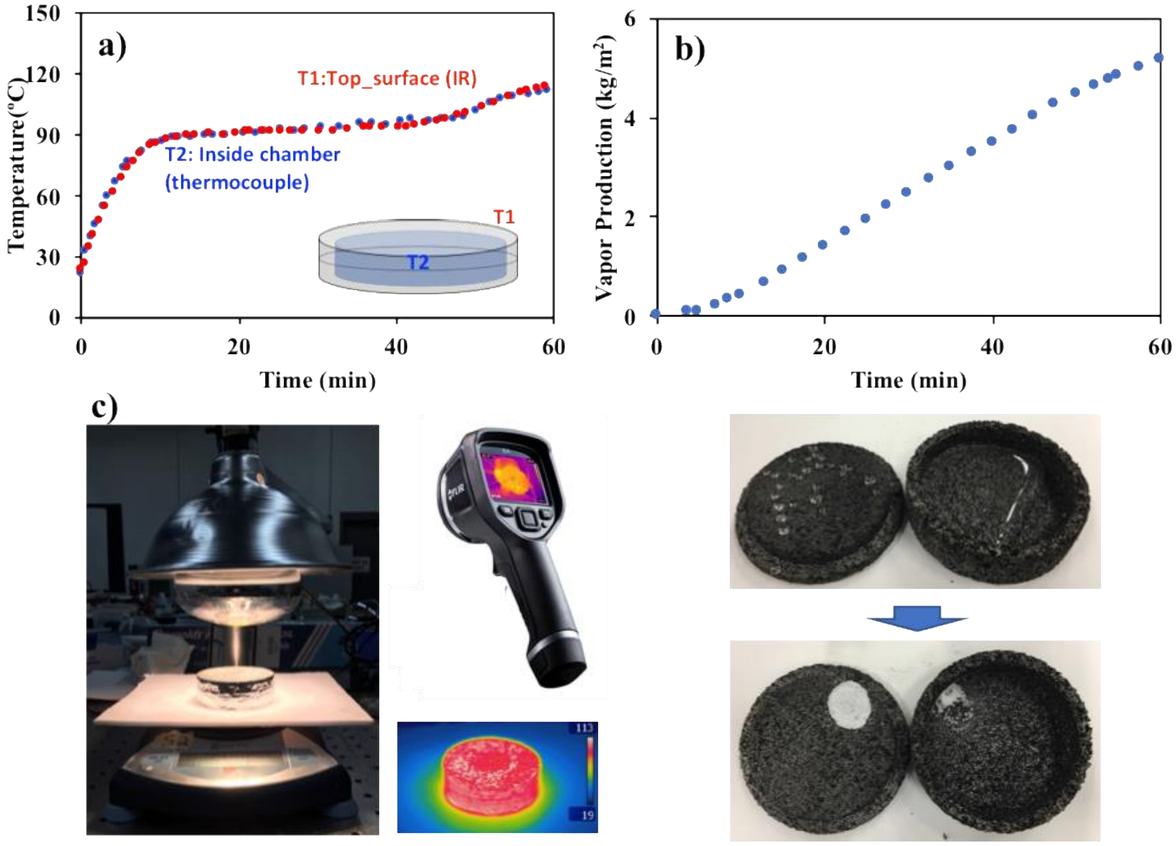

Figure S5. (a) The temperature profile of top surface and inside of GF membrane distillation chamber under concentrated solar-thermal (~estimated 3-sun) irradiation. (b) Evaporation mass loss of salt water. (c) Schematics of experiments and results of solar-thermal desalination.

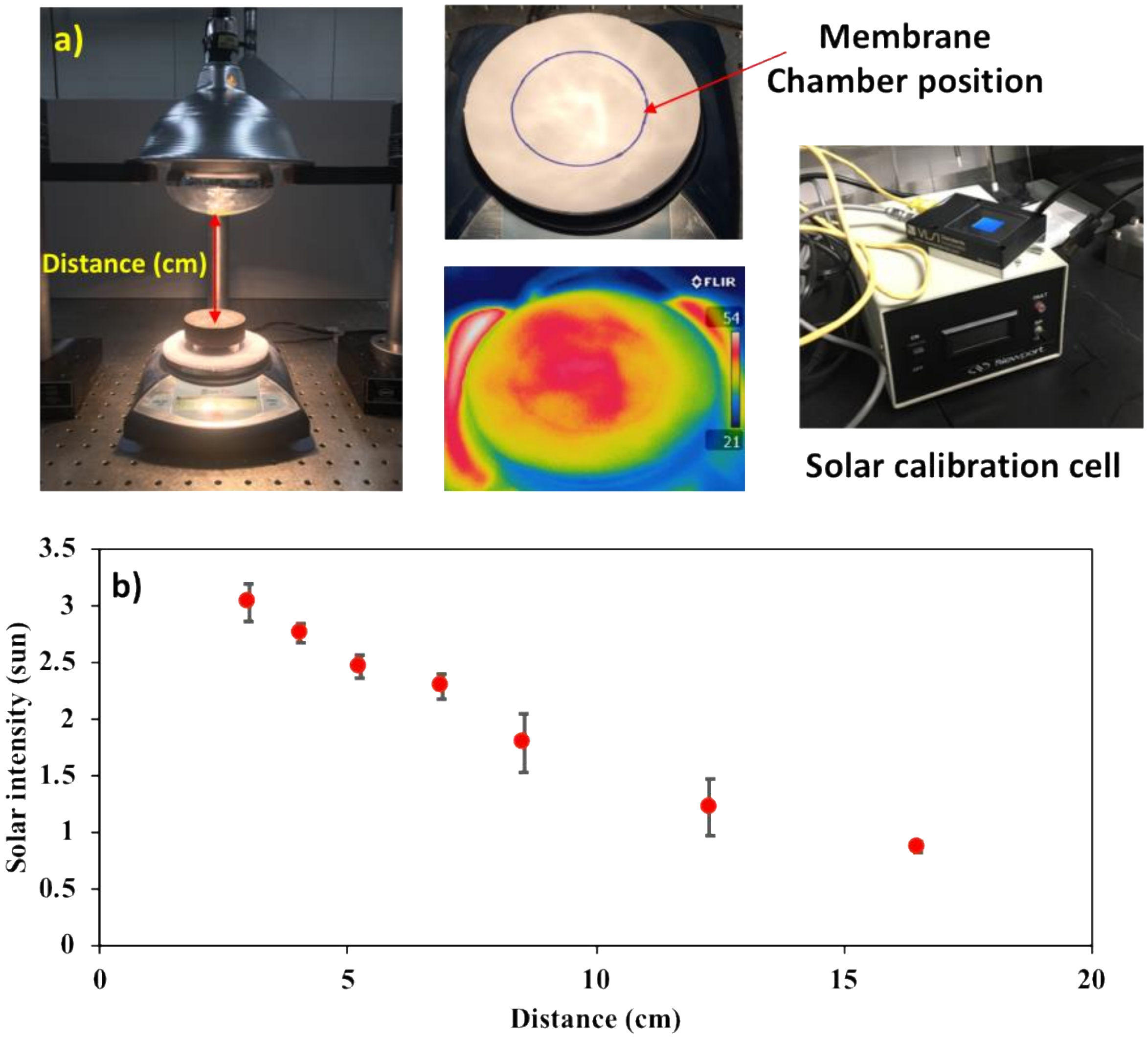

Figure S6. Calibration of concentrated solar-thermal intensity by heat lamp as a function of distance between the bottom surface and membrane. As a result of non-uniform lamp irradiation, the average solar intensity (n=5) in the membrane chamber area was determined by the solar calibration cell.

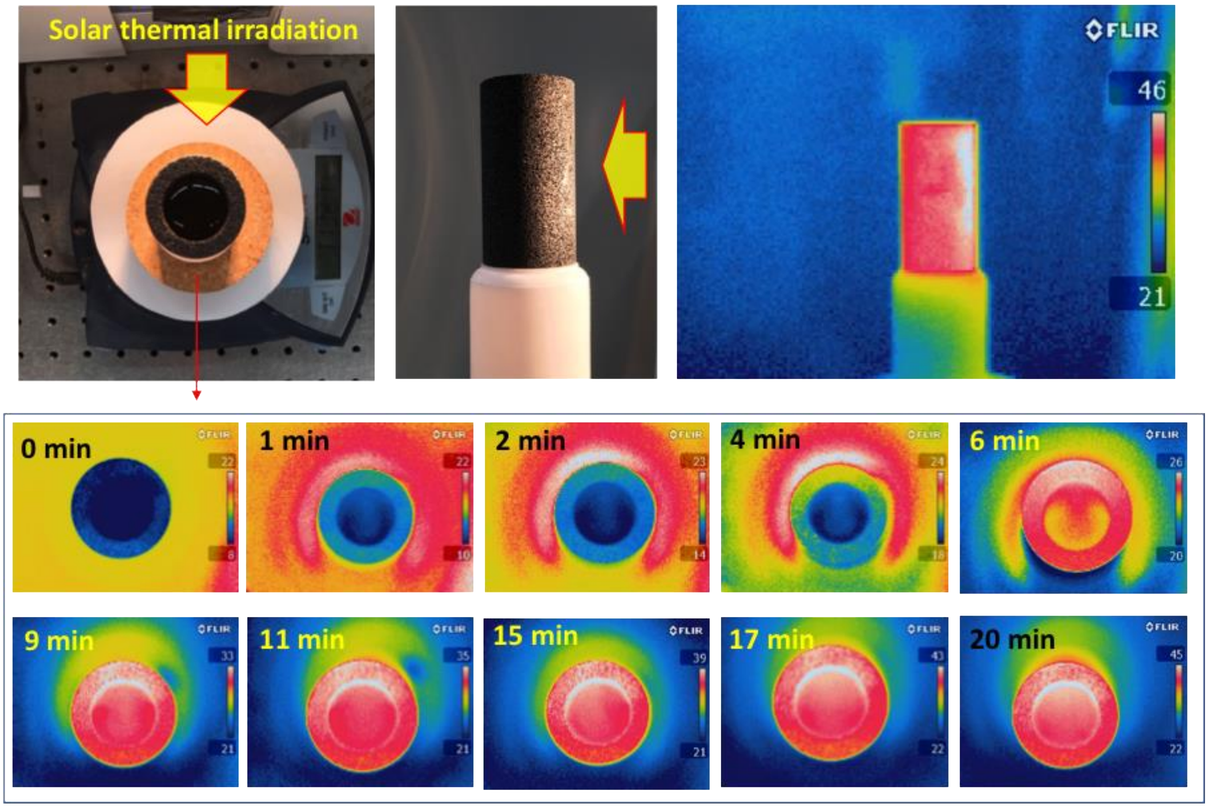

Figure S7. (a) The heat distribution of a tubular GF membrane chamber under concentrated solar-thermal (~ estimated 1-sun) irradiation. The salt water was filled in the membrane chamber.

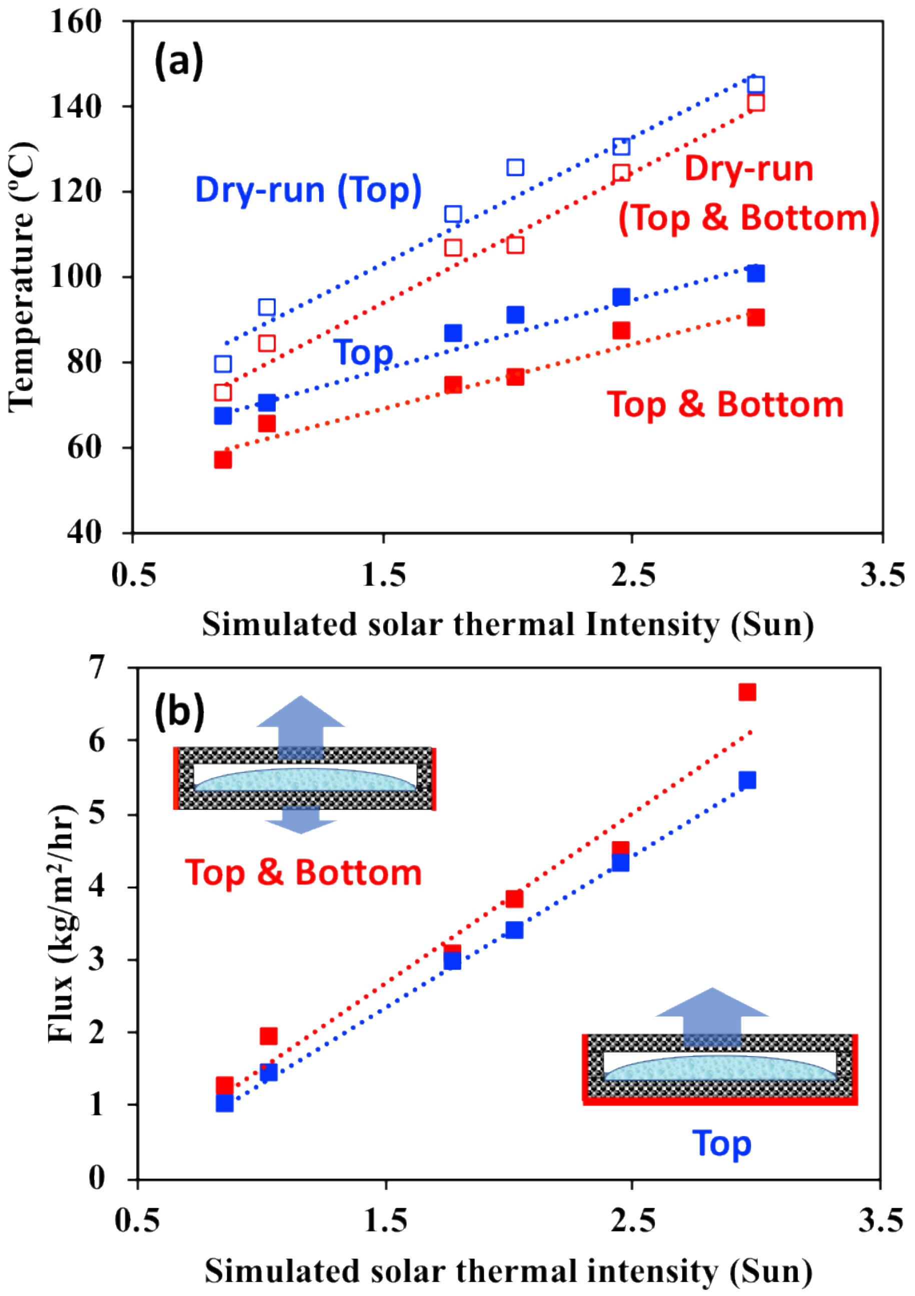

Figure S8. Comparison of desalination performance based on different membrane module system; planar (top) vs. tubular geometry (top and bottom). (a) The temperature rise in the MD chamber under concentrated solar-thermal irradiation. The dry run was the irradiation of the empty membrane chambers. (b) The corresponding mass loss of salt water (3.5 wt % NaCl) under a different solar-thermal irradiation.

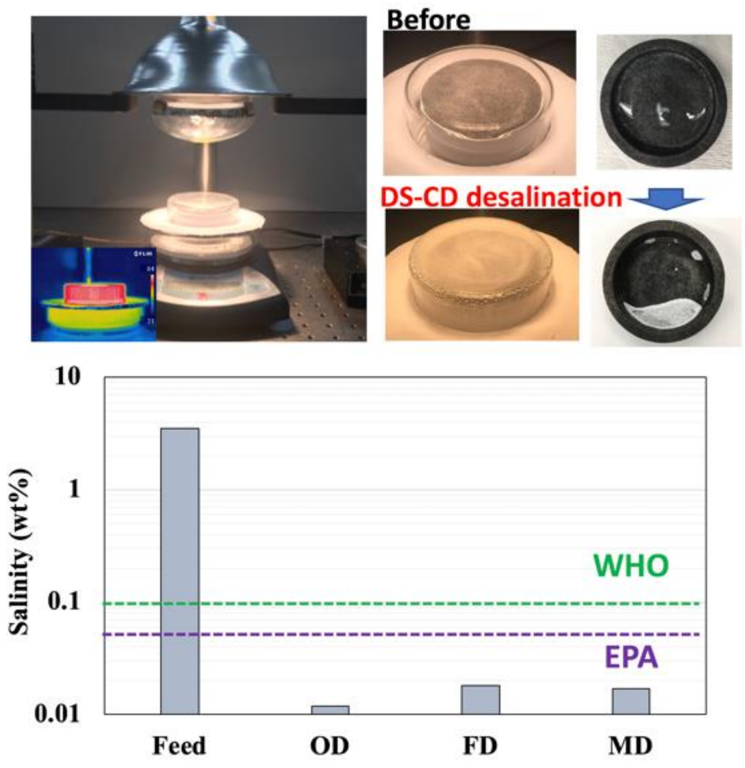

Figure S9. Characteristics of desalination performance based on the different distillation module systems: open-vessel distillation (OD), flow channel distillation (FD), and membrane distillation (MD).

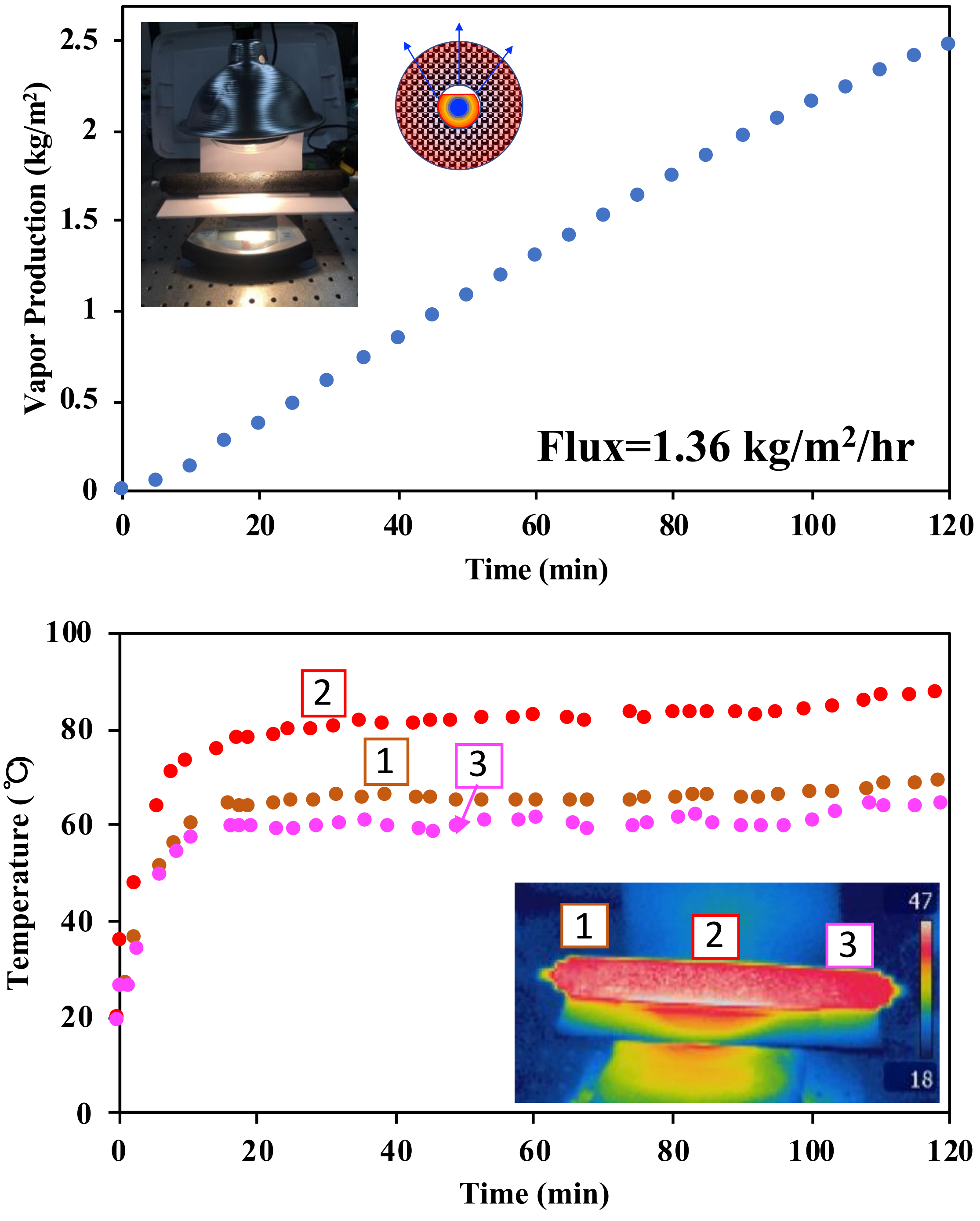

Figure S10. Concentrated solar thermal desalination based on a tubular membrane module.
